# Supplementary material for: Forest edges are globally warmer than interiors and exceed optimal temperatures for vegetation productivity
Source: Commun Earth Environ. 2025 Aug 6;6(1):635. doi: 10.1038/s43247-025-02626-1 (PMC12328228; doi:10.1038/s43247-025-02626-1)
Supplement: Supplementary file 2 — Supplementary Information [file 43247_2025_2626_MOESM2_ESM.pdf]

Supplementary Information

Forest edges are globally warmer than interiors and exceed optimal temperatures for vegetation productivity

Josephine Elena Reek, Thomas W. Crowther, Thomas Lauber, Sebastian Schemm, David Parastatidis, Nektarios Chrysoulakis, Mengtian Huang, Shilong Piao, Constantin M. Zohner, Gabriel Reuben Smith

Contact: josephine.reek@usys.ethz.ch

This file contains:

Supplementary Tables 1-9

Supplementary Figures 1-11

References for Supplementary Information

Supplementary Table 1

Criteria for delineation of seasons

Season	Dates northern hemisphere	Dates southern hemisphere
Spring	06.03.2010 - 24.05.2010	29.05.2010 - 02.12.2010
Summer*	25.05.2010 - 28.08.2010	03.12.2010 - 05.03.2010
Autumn	29.05.2010 - 02.12.2010	06.03.2010 - 24.05.2010
Winter*	03.12.2010 - 05.03.2010	25.05.2010 - 28.08.2010
Wet†	30.03.2010 - 28.03.2010	30.09.2010 - 29.03.2010
Dry†	30.09.2010 - 29.03.2010	30.03.2010 - 28.03.2010

* In order to align the seasons with the solstices the beginning of 2010 and the end of 2010 are grouped together as "winter" (northern hemisphere) and "summer" (southern hemisphere).

† only used for tropics in Supplementary Figures 2 and 6.

Supplementary Table 2

Criteria for delineation of biomes

**Biome (Dinerstein et al., 2017)**[1]	**Our designation**
Tropical and subtropical moist broadleaf forests	tropical
Tropical and subtropical dry broadleaf forests	tropical
Tropical and subtropical coniferous forests	tropical
Temperate broadleaf and mixed forests	temperate
Temperate conifer forests	temperate
Boreal forests or taiga	boreal
Tropical and subtropical grasslands, savannas, and shrublands	tropical
Temperate grasslands, savannas, and shrublands	temperate
Flooded Grasslands and Savannas	other
Montane grasslands and shrublands	other
Tundra	boreal
Mediterranean forests, woodlands, and scrub	temperate
Deserts and xeric shrublands	other
Mangroves	other

Supplementary Table 3

Statistics used for the individual BAMs used in Figure 1. Moran's I values for the residuals of these models are documented in Supplementary Table 8.

Intercept:

Model	Estimate	Standard Error	t value	p value	n
**Spring global**	16.708	0.141	118.608	0	2,135,932
**Summer global**	20.698	0.090	229.819	0	4,125,462
**Autumn global**	12.060	0.110	109.553	0	3,099,187
**Winter global**	7.440	0.190	39.152	0	1,704,217
**Spring tropical**	29.934	0.185	161.819	0	729,889
**Summer tropical**	25.345	0.204	124.351	0	655,721
**Autumn tropical**	22.257	0.168	132.253	0	793,467
**Winter tropical**	22.833	0.133	172.106	0	1,135,746
**Spring temperate**	12.561	0.148	85.146	0	1,098,224
**Summer temperate**	23.230	0.099	234.167	0	1,964,200
**Autumn temperate**	11.108	0.127	87.767	0	1,553,499
**Winter temperate**	-2.486	0.196	-12.659	1.013 E-36	470,157
**Spring boreal**	9.789	0.352	27.839	2.895 E-170	222,391
**Summer boreal**	19.467	0.108	181.042	0	1,381,379
**Autumn boreal**	6.569	0.199	33.003	1.178 E-238	625,444
**Winter boreal**	-15.708	0.506	-31.018	1.364 E-199	8,541

Approximate significance of smooth terms: (Df = estimated degrees of freedom)

Model	Df (Distance)	F value (Distance)	p value (Distance)	Df (SxO)	F value (SxO)	p value (SxO)
**Spring global**	8.974	34,428.472	0	8,957.798	1,133.817	0
**Summer global**	8.993	126,157.560	0	15,618.147	1,037.488	0
**Autumn global**	8.984	52,644.493	0	11,994.684	1,645.189	0
**Winter global**	8.979	47,711.194	0	8,328.883	2,619.078	0
**Spring tropical**	8.934	17,954.017	0	2,638.128	687.066	0
**Summer tropical**	8.856	8,437.744	0	3,016.901	514.252	0
**Autumn tropical**	8.767	5,537.308	0	2,879.228	311.548	0
**Winter tropical**	8.972	41,191.560	0	3,825.552	1,365.897	0
**Spring temperate**	8.937	12,797.876	0	5,217.521	507.942	0
**Summer temperate**	8.990	86,487.520	0	8,112.883	727.810	0
**Autumn temperate**	8.987	60,825.928	0	6,684.551	2,337.999	0
**Winter temperate**	8.922	7,191.113	0	3,755.916	776.083	0
**Spring boreal**	8.773	3,358.666	0	1,156.359	863.690	0
**Summer boreal**	8.983	35,853.664	0	5,031.141	965.517	0
**Autumn boreal**	8.958	14,367.912	0	2,626.297	2,370.082	0
**Winter boreal**	7.878	43.560	0	510.495	169.688	0

Model	Df (Elevation)	F value (Elevation)	p value (Elevation)
**Spring global**	8.845	5,674.813	0
**Summer global**	8.951	16,627.894	0
**Autumn global**	8.749	14,722.050	0
**Winter global**	8.924	16,750.929	0
**Spring tropical**	8.727	2,238.690	0
**Summer tropical**	7.302	92,246.387	0
**Autumn tropical**	7.870	1,466.684	0
**Winter tropical**	8.842	10,395.833	0
**Spring temperate**	8.596	2,377.993	0
**Summer temperate**	8.904	8,588.903	0
**Autumn temperate**	8.958	14.782.336	0
**Winter temperate**	8.868	6,118.352	0
**Spring boreal**	7.391	374.569	0
**Summer boreal**	8.919	1,544.223	0

**Autumn boreal**	7.379	2,721.276	0
**Winter boreal**	8.621	37.667	0

Supplementary Table 4

Statistics for the individual BAMs used in Figure 3, as well as Supplementary Figure 11.

Intercept:

	Estimate	Standard Error	t value	p value	n
**Tropical**	3.795	0.397	9.557	1.233 E-21	101,802
**Temperate**	-0.802	0.142	-5.637	1.731 E-08	451,074
**Boreal**	0.432	0.132	3.270	0.001	494,469

Approximate significance of smooth terms: (Df = estimated degrees of freedom)

	Df (Distance)	F value (Distance)	p value (Distance)	Df (SxO)	F value (SxO)	p value (SxO)
**Tropical**	7.704	139.786	0	439.262	233.580	0
**Temperate**	8.862	2,237.956	0	2,257.614	349.554	0
**Boreal**	8.779	1,638.487	0	1,978.555	582.535	0

	Df (Elevation)	F value (Elevation)	p value (Elevation)
**Tropical**	5.884	16.116	0
**Temperate**	8.102	226.651	0
**Boreal**	7.879	34.905	0

Supplementary Table 5

Statistics for the individual BAMs used in Supplementary Figure 2. Moran's I values for the residuals of these models are documented in Supplementary Table 8.

Intercept:

	Estimate	Standard Error	t value	p value	n
**Wet Season**	27.153	0.130	209.545	0	1,524,045
**Dry Season**	20.758	0.107	194.151	0	2,221,577

Approximate significance of smooth terms: (Df = estimated degrees of freedom)

	Df (Distance)	F value (Distance)	p value (Distance)	Df (SxO)	F value (SxO)	p value (SxO)
**Wet Season**	8.945	21,865.229	0	7,421.894	408.129	0
**Dry Season**	8.978	51,978.936	0	8,458.033	1,136.017	0

	Df (Elevation)	F value (Elevation)	p value (Elevation)
**Wet Season**	8.645	3,629.406	0
**Dry Season**	8.847	12,715.582	0

Supplementary Table 6

Statistics for the BAM used in Supplementary Figure 6.

Intercept:

	Estimate	Standard Error	t value	p value	n
**Wet Season**	3.809	0.252	15.105	1.578 E-51	301,407

Approximate significance of smooth terms: (Df = estimated degrees of freedom)

	Df (Distance)	F value (Distance)	p value (Distance)	Df (SxO)	F value (SxO)	p value (SxO)
**Wet Season**	8.460	672.225	0	1,511.568	296.813	0

	Df (Elevation)	F value (Elevation)	p value (Elevation)
**Wet Season**	6.390	90.483	0

Supplementary Table 7

Statistics for the BAM used in Supplementary Figure 7.

Intercept:

	Estimate	Standard Error	t value	p value	n
**Full year**	4.911	0.162	30.239	1.062 E-200	565,494

Approximate significance of smooth terms: (Df = estimated degrees of freedom)

	Df (Distance)	F value (Distance)	p value (Distance)	Df (SxO)	F value (SxO)	p value (SxO)
**Full year**	8.716	1,320.503	0	3,359.486	278.946	0

	Df (Elevation)	F value (Elevation)	p value (Elevation)
**Full year**	8.801	284.543	0

Supplementary Table 8

Moran's I values for the residuals of the BAMs used in Figure 1 and Supplementary Figure 2. All calculations of Moran's I were performed on a randomly sampled subset of 75,000 datapoints, except for boreal winter, which includes all 8,541 in that dataset.

Model	Observed	Expected	Standard Deviation	p value
Figure 1 models:				
**Spring global**	0.0024	-1.3334 E-05	9.3977 E-05	0
**Summer global**	0.0022	-1.3334 E-05	7.9662 E-05	0
**Autumn global**	0.0019	-1.3334 E-05	8.5650 E-05	0
**Winter global**	0.0047	-1.3334 E-05	9.9473 E-05	0
**Spring tropical**	0.0050	-1.3334 E-05	0.0001	0
**Summer tropical**	0.0062	-1.3334 E-05	0.0001	0
**Autumn tropical**	0.0048	-1.3334 E-05	0.0001	0
**Winter tropical**	0.0063	-1.3334 E-05	0.0001	0
**Spring temperate**	0.0021	-1.3334 E-05	0.0001	0
**Summer temperate**	0.0036	-1.3334 E-05	9.7218 E-05	0
**Autumn temperate**	0.0030	-1.3334 E-05	9.1254 E-05	0
**Winter temperate**	0.0062	-1.3334 E-05	0.0001	0
**Spring boreal**	0.0075	-1.3334 E-05	9.1543 E-05	0
**Summer boreal**	0.0025	-1.3334 E-05	7.6741 E-05	0
**Autumn boreal**	0.0041	-1.3334 E-05	7.9548 E-05	0
**Winter boreal**	0.0268	-0.0001	0.0012	0
Supplementary F2 models:				
**Wet season**	0.0042	-1.3334 E-05	0.0001	0
**Dry season**	0.0031	-1.3334 E-05	0.0001	0

Supplementary Table 9

Statistics for the BAMs used in Supplementary Figure 9.

Intercept:

Model	Estimate	Standard Error	t value	p value	n
**Spring global**	16.897	0.138	122.473	0	2,508,092
**Summer global**	21.079	0.088	238.470	0	4,804,077
**Autumn global**	12.575	0.108	116.210	0	3,612,808
**Winter global**	7.343	0.186	39.557	0	2,003,819
**Spring tropical**	29.985	0.185	162.020	0	853,135
**Summer tropical**	25.464	0.206	123.822	0	765,642
**Autumn tropical**	22.565	0.169	133.344	0	922,152
**Winter tropical**	22.772	0.132	172.158	0	1,319,607
**Spring temperate**	12.742	0.147	86.460	0	1,297,155
**Summer temperate**	23.144	0.099	232.683	0	2,316,657
**Autumn temperate**	11.431	0.127	89.812	0	1,819,565
**Winter temperate**	-2.404	0.196	-12.275	1.252 E-34	548,541
**Spring boreal**	10.073	0.453	22.222	2.743 E-109	238,772
**Summer boreal**	19.625	0.169	116.273	0	1,546,106
**Autumn boreal**	7.073	0.241	29.345	3.624 E-189	694,505
**Winter boreal**	-13.591	0.515	-26.373	4.064 E-148	10,306

Approximate significance of smooth terms: (Df = estimated degrees of freedom)

Model	Df (Distance)	F value (Distance)	p value (Distance)	Df (SxO)	F value (SxO)	p value (SxO)
**Spring global**	8.995	40,669.498	0	9,237.251	1,242.478	0
**Summer global**	8.999	152,789.038	0	15,983.740	1,176.025	0
**Autumn global**	8.997	55,347.852	0	12,336.933	1,599.159	0
**Winter global**	8.997	58,128.659	0	8,666.750	2,905.259	0
**Spring tropical**	8.989	21,793.936	0	2,679.828	775.373	0
**Summer tropical**	8.977	10,441.184	0	3,081.352	588.598	0
**Autumn tropical**	8.960	5,673.435	0	2,923.942	305.724	0
**Winter tropical**	8.996	51,965.86	0	3,891.762	1,542.435	0
**Spring temperate**	8.985	14,565.633	0	5,331.645	586.834	0
**Summer temperate**	8.998	104005.505	0	8,236.902	825.274	0
**Autumn temperate**	8.997	70,095.970	0	6,806.985	2,630.729	0
**Winter temperate**	8.982	7,745.452	0	3,880.135	883.772	0
**Spring boreal**	8.913	3,707.611	0	1,182.628	920.475	0
**Summer boreal**	8.997	41,483.178	0	5,066.741	1,088.792	0
**Autumn boreal**	8.991	16,131.765	0	2,660.850	2,593.563	0
**Winter boreal**	8.572	40.362	0	538.106	181.188	0

Model	Df (Elevation)	F value (Elevation)	p value (Elevation)
**Spring global**	8.911	6,133.630	0
**Summer global**	8.975	20,352.454	0
**Autumn global**	8.783	14,381.579	0
**Winter global**	8.958	17,797.361	0
**Spring tropical**	8.790	2,368.049	0
**Summer tropical**	7.287	2,568.274	0
**Autumn tropical**	7.864	1,318.346	0
**Winter tropical**	8.901	11,569.535	0
**Spring temperate**	8.604	2,543.230	0
**Summer temperate**	8.931	10,162.794	0
**Autumn temperate**	8.970	15,927.593	0
**Winter temperate**	8.933	6,437.692	0
**Spring boreal**	8.267	393.545	0
**Summer boreal**	8.888	1,734.767	0
**Autumn boreal**	8.623	2,606.678	0
**Winter boreal**	8.578	45.559	0

Supplementary Figure 1

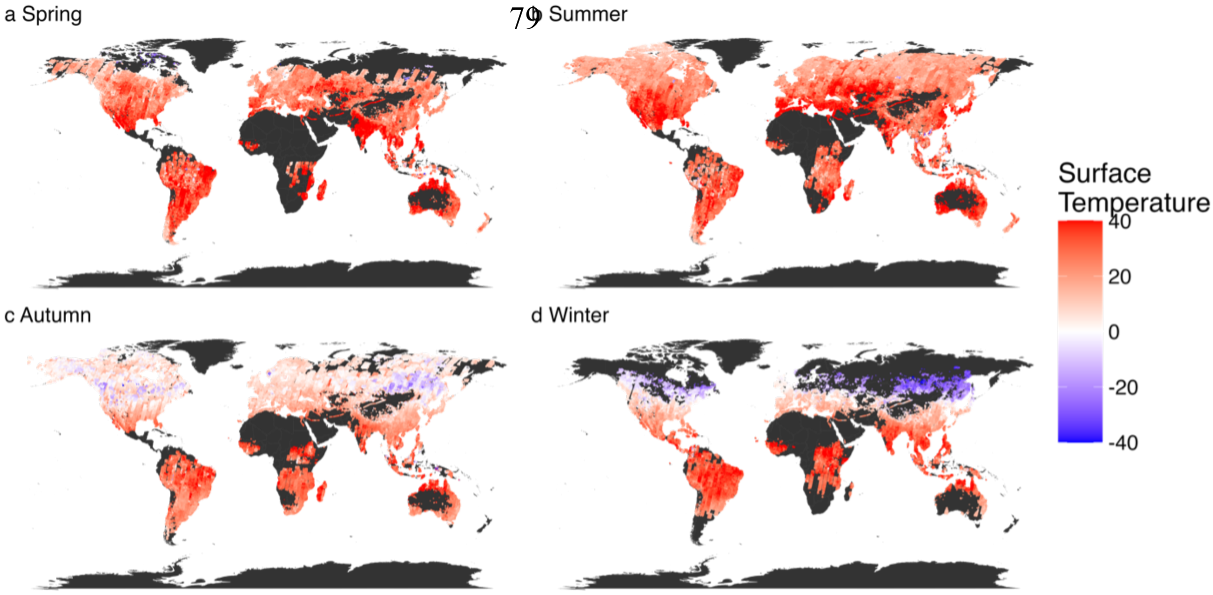

Supplementary Figure 1: T_{surf} data [°C] retrieved by season; **a** spring (n=2,514,909) **b** summer (n=4,820,772) **c** autumn (n=3,624,439) **d** winter (n=2,008,998).

Supplementary Figure 2

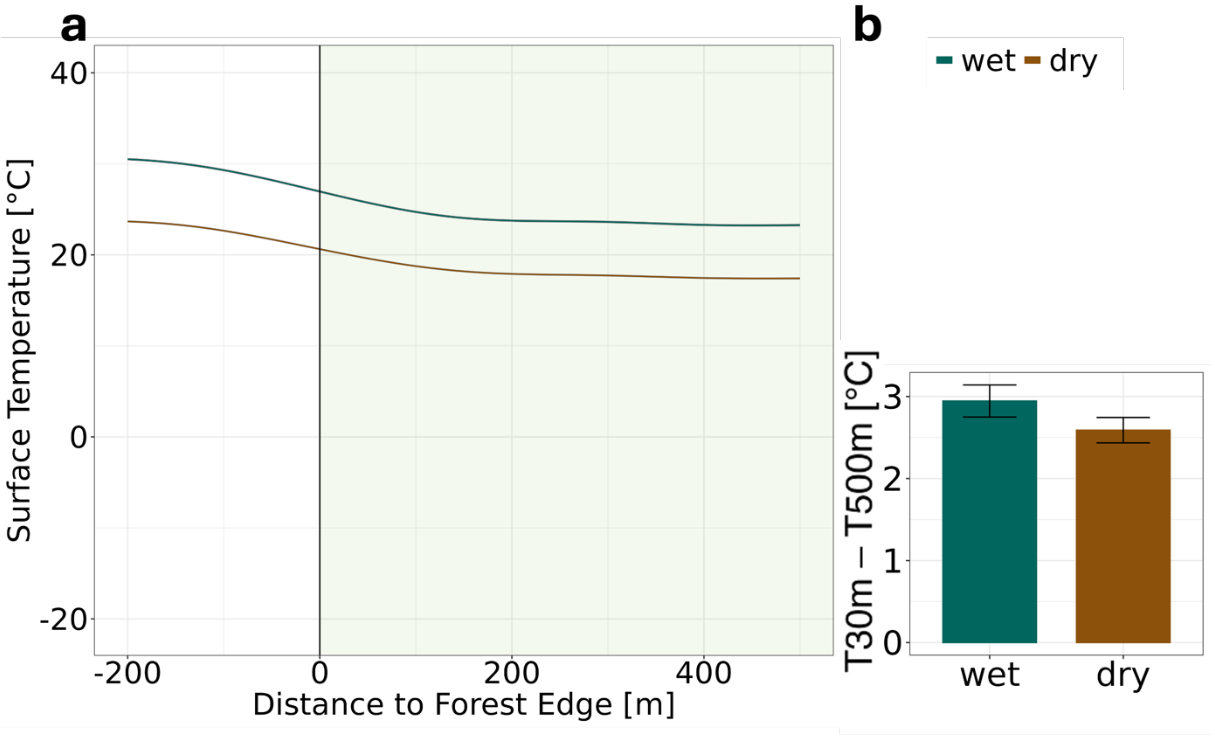

Supplementary Figure 2: Surface Temperature (T_{surf}) around forest edges corrected for satellite scene and overpass, as well as elevation; only tropical biome using wet season and dry season; blue - wet, brown – dry, grey – standard error, green shading – distances inside the forest; **a** curves of T_{surf} across forest edges; **b** difference in T_{surf} between the edge (30m) and the forest interior (500m), error bars denote standard error. Predicted lines based on BAMs with wet season (n=1,524,045), dry season (n=2,221,577). For criteria for delineation of seasons see Supplementary Table 1, delineation of biomes, see SupplementaryTable 2. Statistics for the BAMs are documented in Supplementary Table 5.

Supplementary Figure 3

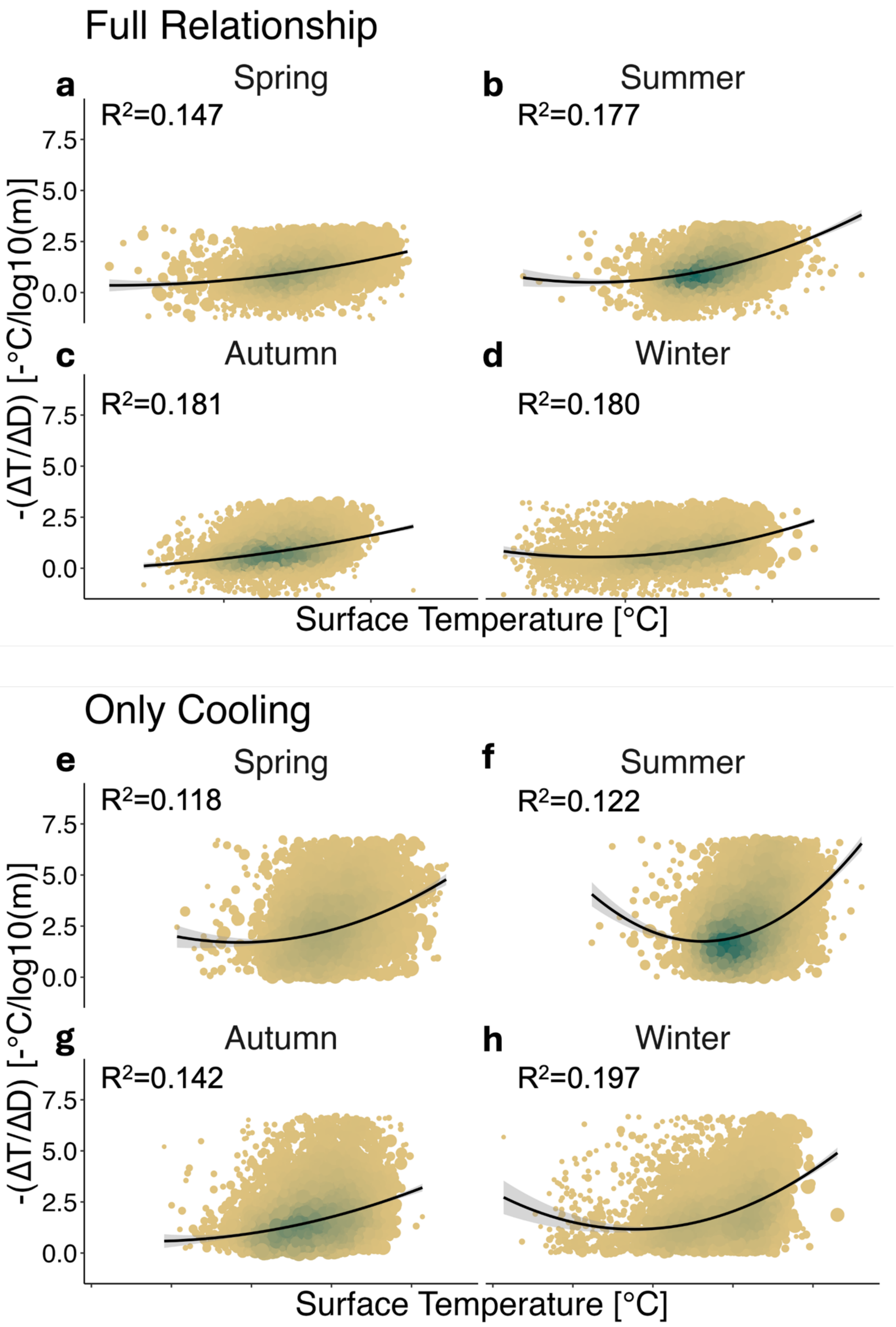

Supplementary Figure 3: Relationship between forest's cooling edge effect and T_{surf}; **a** all locations **b** only locations inside the forest where cooling increased towards the forest interior. Each point corresponds to the strength of the temperature edge effect in one satellite scene (Methods); Black line: quadratic curve weighted by inverse variance of the strength of the edge effect in each satellite scene; Blue – high point density, brown – low point density; larger points correspond to higher weight for the curve fit. a: n=7,752, b: n=13,856, c: n=10,900, d: n=6,947, e: n= 5,398, f: n= 10,302, g: n= 7,878, h: n= 4,563.

Supplementary Figure 4

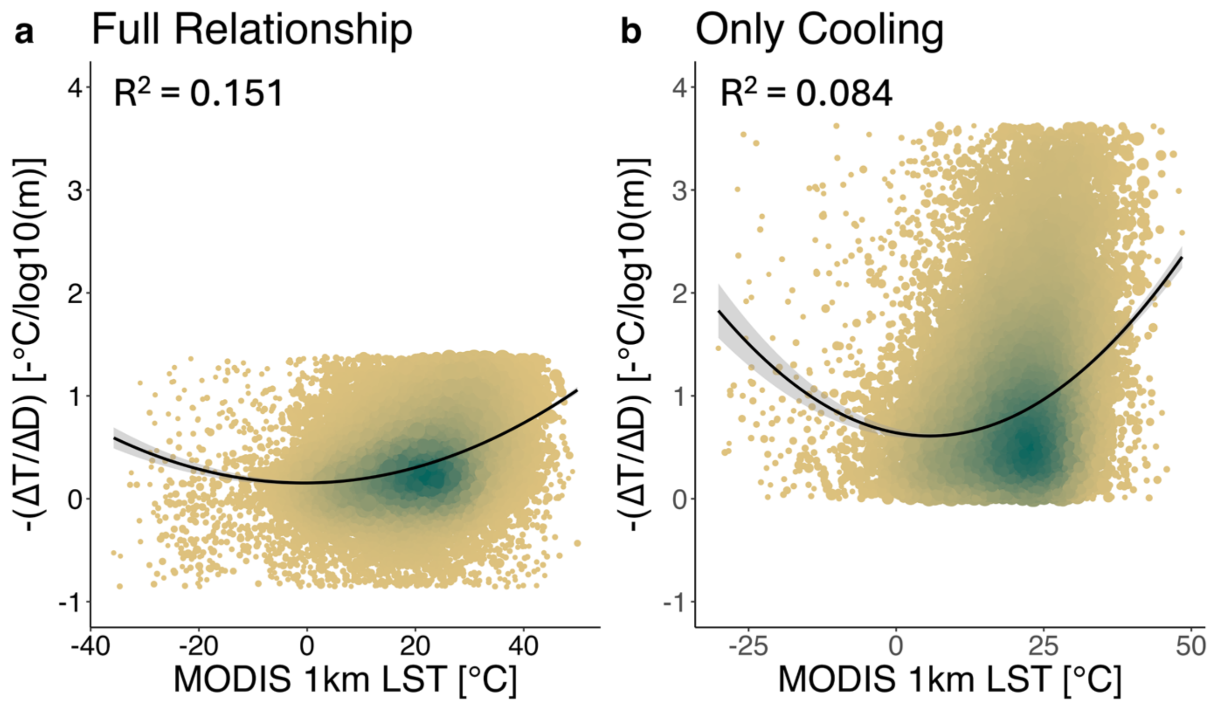

Supplementary Figure 4: Relationship between forest's cooling edge effect and T_{surf} using MODIS satellite 1km LST measurements; **a** all locations (n=29,134) **b** only locations inside the forest where cooling increased towards the forest interior (n=16,628). Each point corresponds to the strength of the temperature edge effect in one satellite scene (Methods); Black line: quadratic curve weighted by inverse variance of the strength of the edge effect in each satellite scene; Blue – high point density, brown – low point density; larger points correspond to higher weight for the curve fit.

Supplementary Figure 5

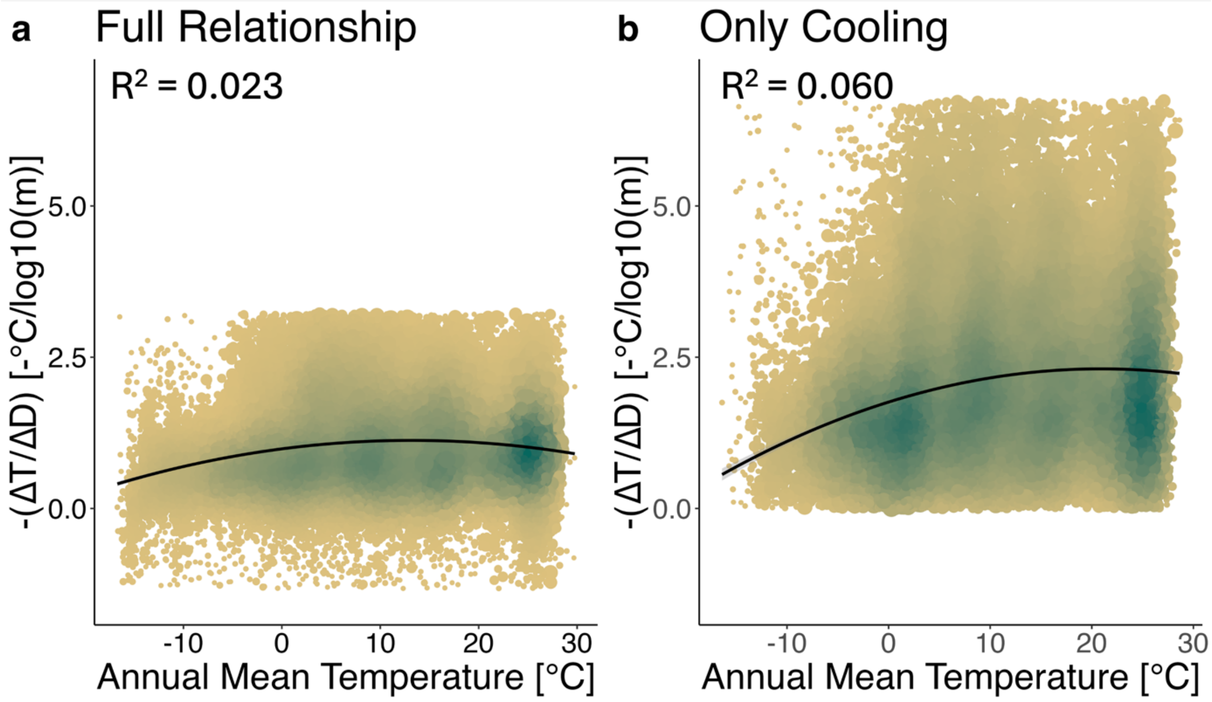

Supplementary Figure 5: Relationship between forest's cooling edge effect and temperature calculating edge effects with Landsat 30m T_{surf} and relating to CHELSEA mean annual temperature; **a** all locations (n=39,441) **b** only locations inside the forest where cooling increased towards the forest interior (n=28,140). Each point corresponds to the strength of the temperature edge effect in one satellite scene (Methods); Black line: quadratic curve weighted by inverse variance of the strength of the edge effect in each satellite scene; Blue – high point density, brown – low point density; larger points correspond to higher weight for the curve fit.

Supplementary Figure 6

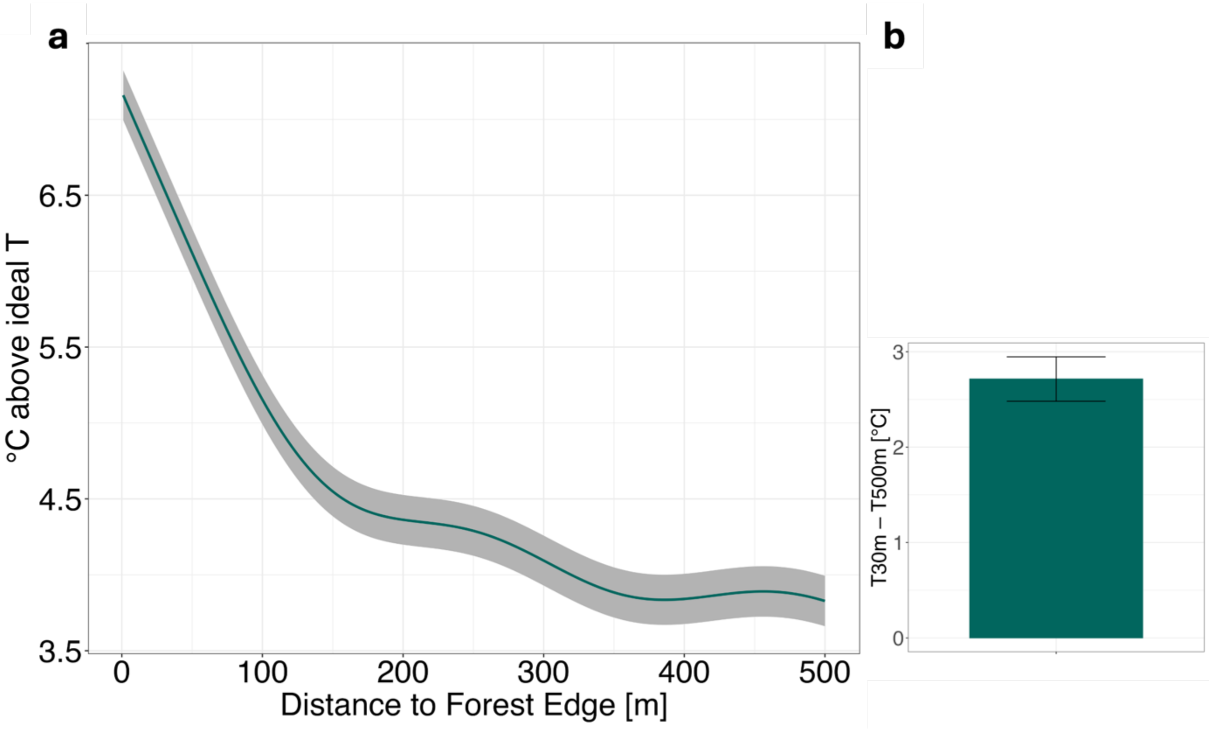

Supplementary Figure 6: Average difference of observed T_{surf} - ideal T_{surf} for ecosystem level productivity in the tropics during wet season. grey/ error bars denote standard error; **a** °C above ideal temperature for productivity with distance to forest edge; **b** difference of °C above ideal at forest edge (30m) and forest interior (500m). Predicted line based on BAM with n=301,407. Statistics for the BAM are documented in Supplementary Table 6.

Supplementary Figure 7

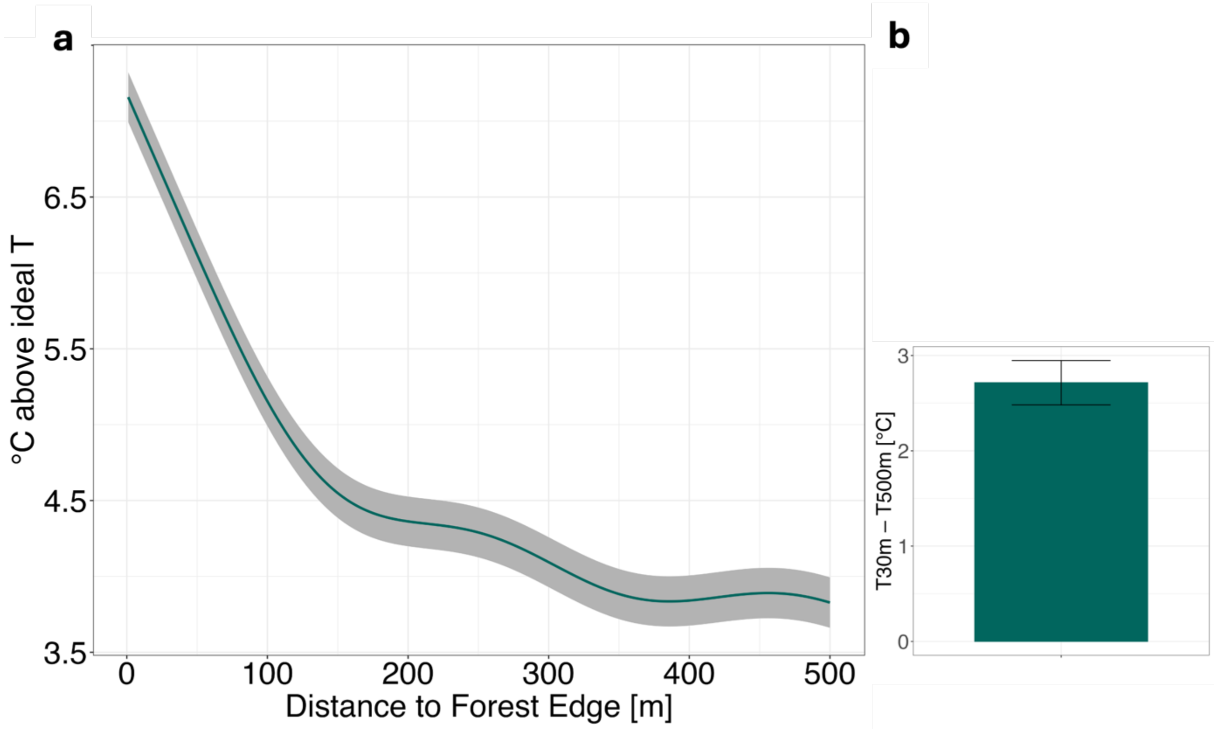

Supplementary Figure 7: Average difference of observed T_{surf} - ideal T_{surf} for ecosystem level productivity in Tropical and subtropical moist broadleaf forests during the full year, as these regions contain evergreen forests with less seasonal variation in productivity. "Tropical and subtropical moist broadleaf forests" are defined according to Supplementary Table 2. grey/ error bars denote standard error; **a** °C above ideal temperature for productivity with distance to forest edge; **b** difference of °C above ideal at forest edge (30m) and forest interior (500m); Predicted line based on BAM with n=565,494. Statistics for the BAM are documented in Supplementary Table 7.

Supplementary Figure 8

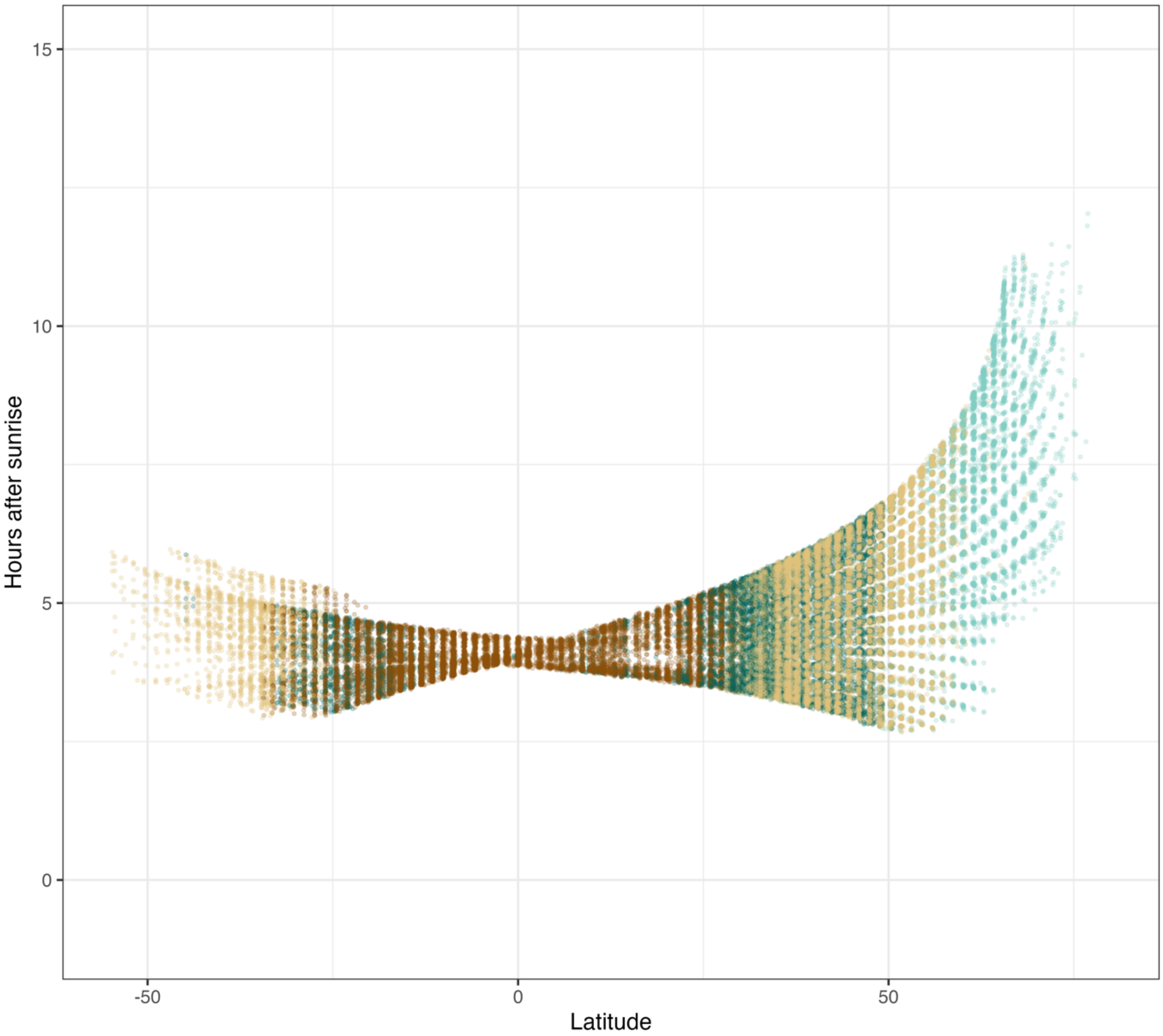

Supplementary Figure 8: Hours after sunrise at which satellite scenes were captured n = 49,906; light blue – boreal, beige – temperate, brown – tropical, dark blue – other; 770 SxO in the boreal biome are not depicted as no sunrise could be calculated (see methods).

Supplementary Figure 9

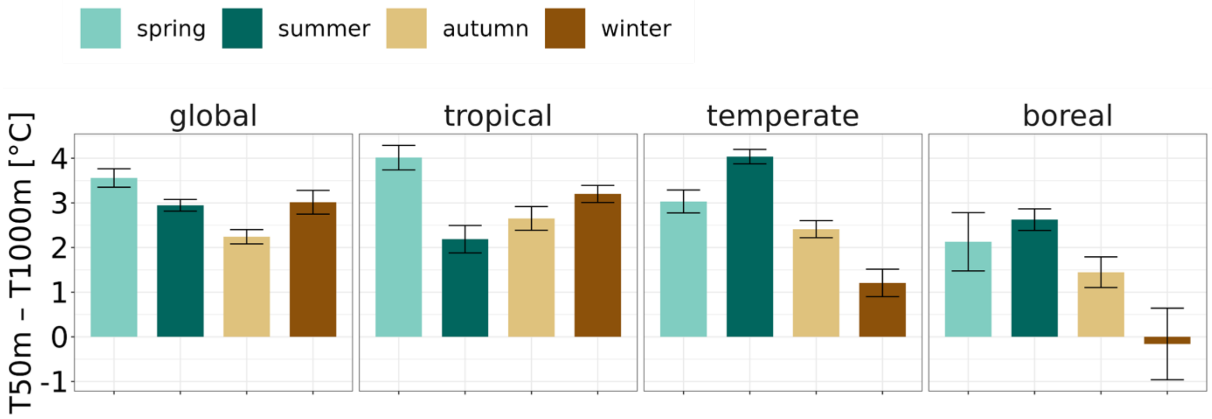

Supplementary Figure 9: Difference in T_{surf} between the edge (50m) and the forest interior (1'000m) corrected for satellite scene and overpass as well as elevation; Using predictions based on BAMs with global (n=12,928,796), tropical (n=3,860,536), temperate (n=5,981,918), boreal (n=2,489,689) analogous to Figure 1. Error bars denote standard error. For criteria for delineation of seasons see Supplementary Table 1, delineation of biomes, see Supplementary Table 2. Statistics for the BAMs are documented in Supplementary Table 9.

Supplementary Figure 10

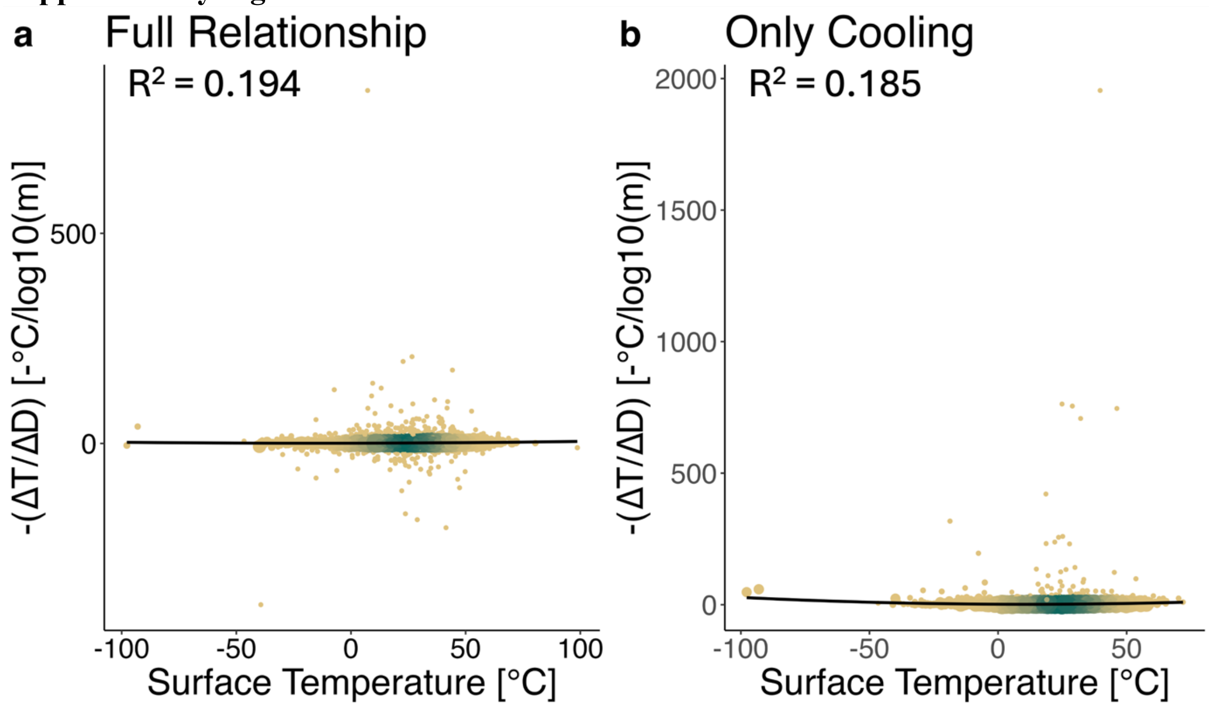

Supplementary Figure 10: Relationship between forest's cooling edge effect and T_{surf} without removing outliers **a** all locations (n=43,268) **b** only locations inside the forest where cooling increased towards the forest interior (n=29,953); Each point corresponds to the strength of the temperature edge effect in one satellite scene (Methods); Black line: quadratic curve weighted by inverse variance of the strength of the edge effect in each satellite scene; Blue – high point density, brown – low point density; larger points correspond to higher weight for the curve fit.

Supplementary Figure 11

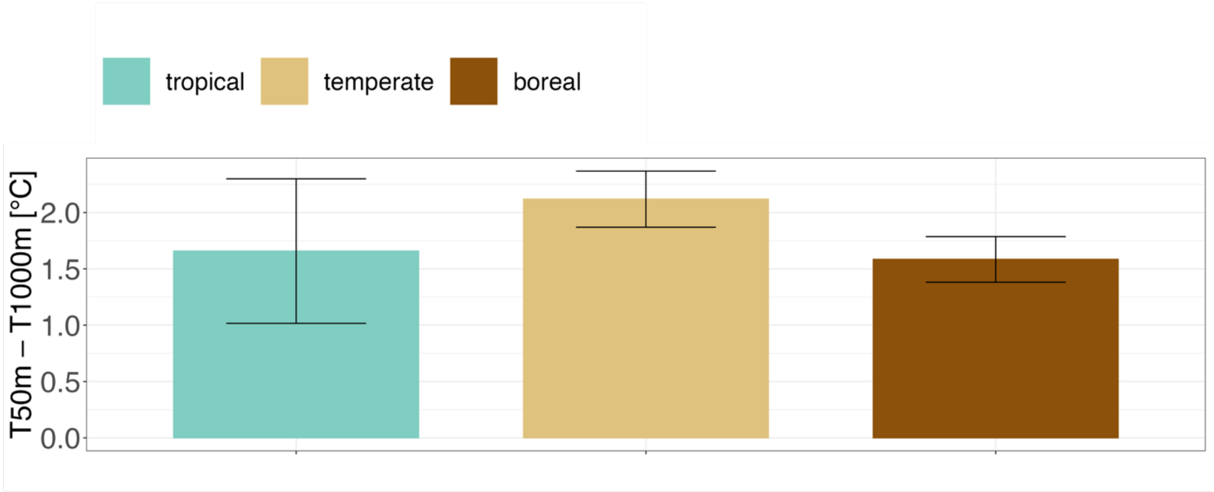

Supplementary Figure 11: Difference of °C above ideal at forest edge (50m) and forest interior (1000m) corrected for satellite scene and overpass as well as elevation; Using predictions based on BAMs with tropical (n=101,803), tropical (n=451,074), boreal (n=494,469) analogous to Figure 3. Error bars denote standard error. For criteria for delineation of biomes, see Supplementary Table 2. Statistics for the BAMs are documented in Supplementary Table 4.

References

1. Dinerstein, E. *et al.* An Ecoregion-Based Approach to Protecting Half the Terrestrial Realm. *BioScience* **67**, 534–545 (2017).
